# Double *CHEK2* Pathogenic and Low-Risk Variants and Associated Cancer Phenotypes

**DOI:** 10.1001/jamanetworkopen.2024.51361

**Published:** 2025-01-02

**Authors:** Brittany L. Bychkovsky, Nihat B. Agaoglu, Carolyn Horton, Linda Polfus, Marcy E. Richardson, Colin Young, Rochelle Scheib, Judy E. Garber, Huma Q. Rana

**Affiliations:** 1 Division of Cancer Genetics and Prevention, Dana-Farber Cancer Institute, Boston, Massachusetts; 2 Department of Medical Oncology, Dana-Farber Cancer Institute, Boston, Massachusetts; 3 Harvard Medical School, Boston, Massachusetts; 4 Department of Medical Genetics, Umraniye Training and Research Hospital, İstanbul, Turkey; 5 Institute of Clinical Cancer Research, Krankenhaus Nordwest, Frankfurt am Main, Germany; 6 Ambry Genetics, Aliso Viejo, California

## Abstract

**Importance:**

*CHEK2* pathogenic and likely pathogenic variants (PVs) are common, and low-risk (LR) variants, p.I157T, p.S428F, and p.T476M, are even more common. Biallelic *CHEK2* PVs are associated with specific cancer phenotypes, including early age at onset of breast cancers. Whether biallelic LR variants are associated with cancer predisposition is unknown.

**Objective:**

To characterize the cancer phenotype among individuals with biallelic *CHEK2* variants, specifically those that have been associated with lower cancer risk in the heterozygous state.

**Design, Setting, and Participants:**

This retrospective observational cohort study examining cancer phenotype by *CHEK2* genotype was conducted at a single diagnostic genetic testing laboratory. Of 36 821 individuals who underwent genetic testing, 3783 (10.3%) with *CHEK2* PVs or LR variants were ascertained from July 1, 2012, to September 30, 2019. Analyses were conducted from September 2022 to January 2024.

**Exposures:**

Cancer phenotype among individuals with 2 LR variants and those with 1 PV and 1 LR variant was compared with cancer phenotype among individuals with wild type (WT) (n = 33 034), single LR variant (n = 1566), single PV controls (n = 2167), and 2 PVs (n = 21). Cancer phenotypes were investigated for any cancer, multiple primary cancers, female breast cancer, and bilateral female breast cancers.

**Main Outcomes and Measures:**

Cancer phenotype of CHEK2 2 LRs and 1 PV and 1 LR.

**Results:**

Of 36 821 individuals, 92.1% were female, and the median age at testing was 53 years (IQR, 44-63 years); 3787 (10.3%) were identified as having a *CHEK2* PV or LR variant. There were 13 individuals with 2 LR variants and 20 with 1 PV and 1 LR variant. Among those with 2 LR variants, prevalence of any cancer (76.9%) and breast cancer (60.0%) were similar to those with WT (any cancer, 69.8%; breast cancer, 52.7%) and those with a single LR variant (any cancer, 70.9%; breast cancer, 57.5%). Among participants with 1 PV and 1 LR variant, 95.0% had a prior cancer diagnosis, a higher rate than among those with a single PV (76.8%), but the difference was not statistically significant. Among female individuals with 1 PV and 1 LR variant, 86.7% had a breast cancer diagnosis, compared with 67.1% with a single PV, although these differences were not statistically significant.

**Conclusions and Relevance:**

In this cohort study, individuals with 2 LR variants in *CHEK2* had a cancer phenotype similar to those with a single LR variant and similar to WT controls. Individuals with 1 PV and 1 LR variant may have a more penetrant cancer phenotype than individuals with a single PV. Future studies focused on *CHEK2* LR variants will aid in better understanding whether these variants are genetic modifiers associated with cancer risk.

## Key Points

**Question:**

Are *CHEK2* low-risk (LR) variants p.I157T, p.S428F, and p.T476M associated with cancer phenotype when in a biallelic state similar to biallelic pathogenic and likely pathogenic variants (PVs) in *CHEK2*?

**Findings:**

In this cohort study including 3783 individuals with *CHEK2* PVs and LR variants, individuals with 2 LR variants in *CHEK2* had a cancer phenotype similar to those with a single LR variant and wild-type controls. Compared with individuals with 1 PV, a higher percentage of those with 1 PV and 1 LR variant had a prior cancer diagnosis, but the difference was not statistically significant.

**Meaning:**

More studies are needed to understand if LR variants in *CHEK2* are genetic modifiers associated with cancer risk.

## Teaser

This cohort study characterizes the cancer phenotype among individuals with biallelic *CHEK2* variants, specifically variants that have been associated with lower cancer risk in the heterozygous state.

## Introduction

Monoallelic pathogenic and likely pathogenic variants (PVs) in *CHEK2* (OMIM 604373) are associated with a predisposition to breast cancer and have been associated with colorectal, kidney, and thyroid cancers.^1,2^ Little is known about the phenotype associated with biallelic *CHEK2* PVs; however, the phenotype is not known to be associated with severe childhood-onset conditions such as observed with biallelic *ATM* (OMIM 607585) or *BRCA1/2* (*BRCA1*: OMIM 113705; *BRCA2*: OMIM 600185) PVs. Previous studies reported that biallelic *CHEK2* PVs are associated with higher rates of breast cancer and earlier age at onset (median age, 40.5-43.5 years vs 47-49 years for monoallelic).^2,3,4,5^ Biallelic *CHEK2* PVs were also associated with an earlier age at onset of any cancer by a decade (median age, 37 years vs 47 years for monoallelic PVs).^2^

Typically, *CHEK2* PVs are associated with moderate risk for breast cancers, conferring odds ratios (ORs) of 2- to 3-fold. Specific variants in *CHEK2,* such as p.I157T, p.S428F, and p.T476M, have lower ORs (1.1-1.4) and therefore are considered lower risk and have discordant classifications and an ambiguous effect on screening recommendations.^6,7^ Less is known about whether biallelic low-risk (LR) *CHEK2* variants are associated with a specific cancer susceptibility phenotype. One report found that females with biallelic loss-of-function PVs had higher rates of multiple cancers, first cancer diagnosis at a younger age, higher breast cancer rates, and younger age at colorectal cancer diagnosis compared with homozygous p.I157T carriers.^5^ However, the cancer phenotype of p.I157T homozygosity was not compared with monoallelic p.I157T or controls.^5^ These LR variants have a high population frequency, so it is important to discern whether they are associated with cancer predisposition in the biallelic state and, if so, the nature of that predisposition. Given the lack of published data, but high population frequencies of LR variants, we sought to characterize the cancer phenotype of individuals with biallelic LR variants in contrast with PVs and wild type (WT).

## Methods

This retrospective cohort study included individuals with *CHEK2* PVs identified by genetic testing ordered from July 1, 2012, to September 30, 2019, at a single diagnostic testing laboratory (Ambry Genetics, Aliso Viejo, California). Individuals with a PV in another gene were excluded. The reported PV cohort underwent 8- to 25-gene targeted breast and ovarian cancer panel testing (n = 16), 49- to 67-gene panel testing (n = 11), or testing with a customizable panel of 1 to 75 genes (n = 1). The WT cohort included 33 034 individuals without any PVs on a pancancer panel (49-67 genes). Individuals with variants of uncertain significance in any gene were not excluded. The WGC Institutional Review Board (formerly the Western Institutional Review Board) determined the study to be exempt from the Office for Human Research Protections Regulations for the Protection of Human Subjects (45 CFR 46) and provided a waiver of consent for these deidentified data. This study followed the Strengthening the Reporting of Observational Studies in Epidemiology (STROBE) reporting guideline.

Clinical characteristics including race and ethnicity were obtained from clinical documentation from self-reported fields. Race and ethnicity (Asian, Black, Hispanic, and White) categories were defined by investigators based on the US Office of Management and Budget classification of federal data on race and ethnicity and the Institute of Medicine report *Race, Ethnicity, and Language Data Collection: Standardization for Health Care Quality Improvement*.^8^ The “other” race and ethnicity category was defined as those that did not self-identify as Asian, Black, Hispanic, or White from self-reported fields. Given the historic emphasis on Ashkenazi Jewish ancestry as a criteria for cancer genetic testing, we present race and ethnicity for our cohort.

Variant interpretation was based on the American College of Medical Genetics and Genomics/Association for Molecular Pathology guidelines.^9,10^ Pathogenic and likely pathogenic variants were analyzed together. These PVs were categorized as biallelic or monoallelic. Biallelic cases with the same 2 PVs in a given individual were removed to reduce the chance of the variants being on a haplotype rather than in trans. Low-risk *CHEK2* variants included p.I157T (c.470T>C, dbSNP: rs17879961), p.S428F (c.1283C>T, dbSNP: rs137853011), and p.T476M (c.1527C>T, dbSNP: rs142763740).

### Statistical Analysis

Analyses were conducted from September 2022 to January 2024. Descriptive statistics stratified by variant grouping are summarized for categorical characteristics. Frequencies of cancer and ORs with 95% CIs were determined by genotype. All statistical tests were 2-sided, *P* < .05 was considered statistically significant, and analyses were conducted with R, version 4.0.4 (R Project for Statistical Computing). Due to the paucity of male individuals in the sample (n = 8), breast cancer phenotype analysis was restricted to female individuals.

## Results

**Figure 1.**
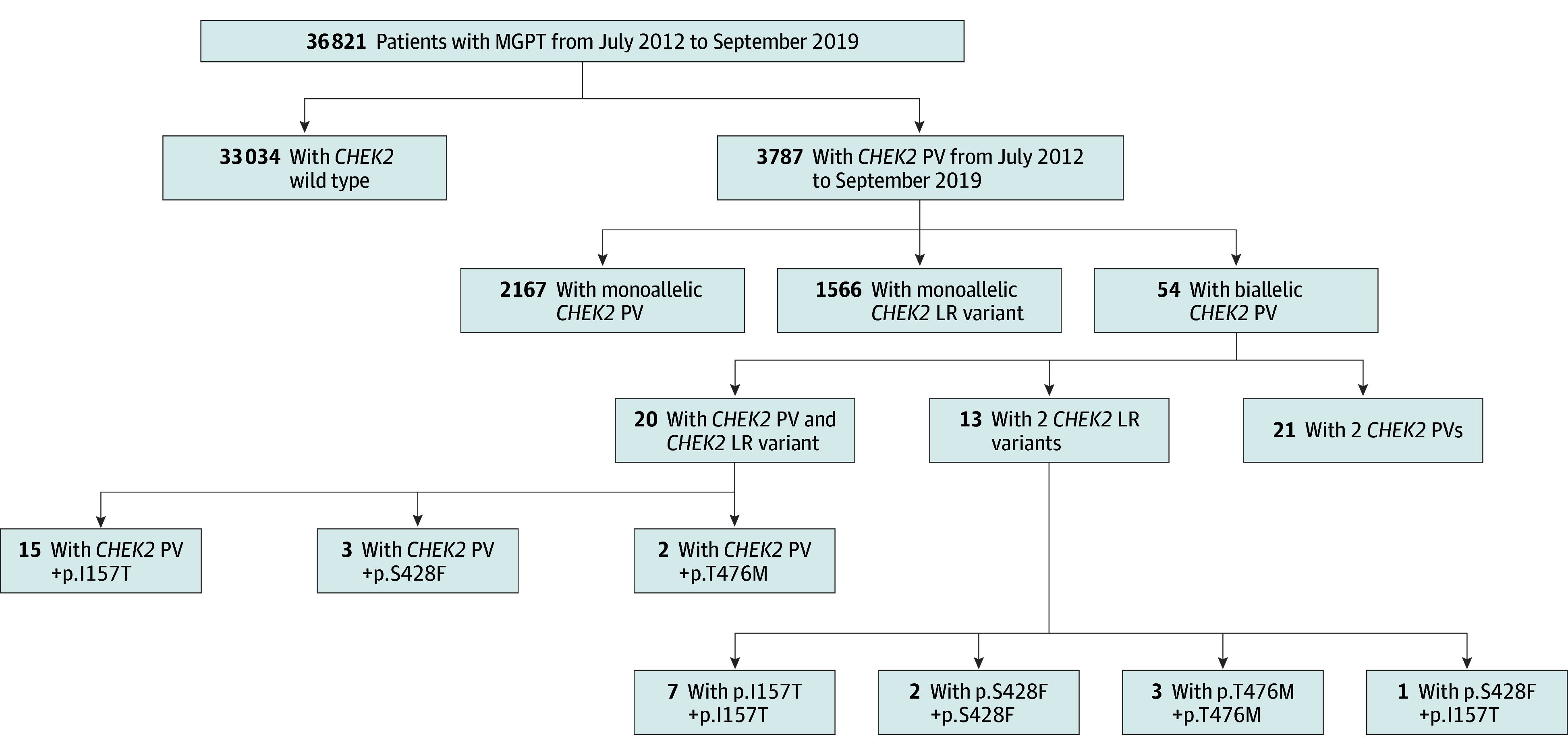
Distribution of *CHEK2* Variants in Study Population LR indicates low-risk; MGPT, multigene panel testing; and PV, pathogenic and likely pathogenic variant.

Among the 36 821 individuals who underwent large panel genetic testing in our cohort, the median age at testing was 53 years (IQR, 44-63 years); 92.1% were female individuals and 7.9% were male individuals; and 7.1% were African American or Black, 5.1% were Ashkenazi Jewish, 4.5% were Asian, 3.0% were Hispanic, 67.2% were White, and 13.1% were other race or ethnicity (those who identified as not African American or Black, Ashkenazi Jewish, Asian, Hispanic, or White) (eTable 1 in Supplement 1). Of the 36 821 individuals, 3787 (10.3%) were identified with a *CHEK2* PV or LR variant, and 54 harbored biallelic *CHEK2* variants (Figure 1). The distribution among 54 individuals with biallelic variants was 20 with PV and LR variant, 21 with 2 PVs, and 13 with 2 LR variants (Figure 1). Demographic information for these participants (ie, 2167 participants with a monoallelic PV and 1566 participants with a monoallelic LR variant( are summarized in eTable 1 in Supplement 1.

### Cancer Phenotype Comparing 2 LR Variants, PV and LR Variant, and 2 PVs vs WT and Monoallelic LR Variant or PV Controls

**Figure 2.**
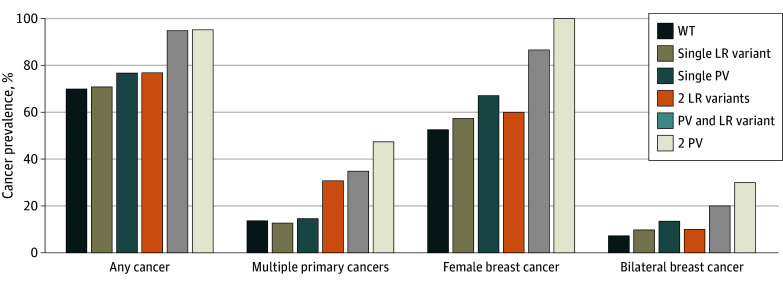
Cancer Prevalence by *CHEK2* Variant and Combination LR indicates low-risk; PV, pathogenic and likely pathogenic variant; and WT, wild type.

Comparisons of individuals with biallelic variants in *CHEK2* were made with WT (n = 33 034), as well as LR variant heterozygotes (n = 1566) or PV heterozygotes (n = 2167) for any cancer, multiple primary cancers, breast cancer, and bilateral breast cancer (Figure 2; Table).

**Table.** All Cancer and Female Breast Cancer Prevalence by *CHEK2* Variant and Combination

Cancer category	*CHEK2* variant category, patients identified, No. (%)						PV and LR variant vs single PV	
	WT (n = 33 034)	Single LR variant (n = 1566)	Single PV (n = 2167)	2 LR variants (n = 13)	PV and LR variant (n = 20)	2 PV (n = 21)	OR	*P* value
Any cancer	23 065 (69.8)	1110 (70.9)	1664 (76.8)	10 (76.9)	19 (95.0)	20 (95.2)	5.75	.06
Multiple primary cancers	4567 (13.8)	200 (12.8)	316 (14.6)	4 (30.8)	7 (35.0)	10 (47.6)	2.49	.72
Female patients identified, No.	30 429	1434	1995	10	15	20	NA	NA
Female breast cancer	16 029 (52.7)	824 (57.5)	1339 (67.1)	6 (60.0)	13 (86.7)	20 (100.0)	3.01	.17
Bilateral breast cancer	2228 (7.3)	141 (9.8)	273 (13.7)	1 (10.0)	3 (20.0)	6 (30.0)	1.54	.46

Abbreviations: OR, odds ratio; LR, low-risk; PV, pathogenic and likely pathogenic variant; WT, wild type.

### Any Cancer

Participants with 2 LR variants had similar cancer prevalence (76.9%) compared with WT (69.8%) and those with a monoallelic LR (70.9%) or a monoallelic PV (76.8%) (Table). Among individuals with a PV and LR variant, 95.0% had a prior cancer diagnosis, similar to those with 2 PVs in *CHEK2* (95.2%). Cancers among those with biallelic 2 LR variants and PV and LR variant in *CHEK2* are reported in eTable 3 in Supplement 1.

### Multiple Primary Cancers

Multiple primary cancers were most frequent among individuals with 2 PVs (47.6%) followed by those with PV and LR variant (35.0%), 2 LR variants (30.8%), a monoallelic PV (14.6%), WT (13.8%), and a monoallelic LR variant (12.8%) (Figure 2; Table).

### Breast Cancer

Breast cancer was most frequent among individuals with 2 PVs in *CHEK2* (100%) followed by those with PV and LR variant (86.7%), a monoallelic PV (67.1%), 2 LR variants (60.0%), a monoallelic LR variant (57.5%), and WT (52.7%) (Figure 2; Table). For bilateral breast cancer, the frequencies were highest for individuals with 2 PVs (30.0%), followed by those with PV and LR variant (20.0%), a monoallelic PV (13.7%), 2 LR variants (10.0%), a monoallelic LR variant (9.8%), and WT (7.3%). Odds ratios were calculated by comparing cancer incidence (any cancer, multiple primary cancer, breast cancer, and bilateral breast cancer) among participants with a monoallelic PV in *CHEK2* and those with PV and LR variant, and the differences were not statistically significant (Table). We examined the frequencies of several cancer types without any significant findings.

For breast cancer cases, the hormone receptor subtype of the first breast cancer was reviewed. Most of the breast cancers were estrogen receptor positive and/or progesterone receptor positive, and few were ERBB2 positive (eTable 2 in Supplement 1). None of the breast cancer among patients with 2 LR variants or PV and LR variant were reported to be triple negative.

## Discussion

In this cohort study, the *CHEK2* LR variants p.I157T, p.S428F, and p.T476M were associated with cancer, multiple primary cancers, breast cancer, and bilateral breast cancer when found in combination with a PV, but not in the biallelic state. Individuals with PV and LR variant had a more penetrant cancer phenotype compared with individuals with 1 PV. A prior study showed that LR variants were not associated with breast, kidney, or thyroid cancer like *CHEK2* PVs,^2^ and these findings can inform counseling of individuals with p.I157T, p.S428F, and p.T476M in regard to their personal cancer risk. Here, we show that *CHEK2* LR variants are likely associated with a modified cancer phenotype when individuals also harbor a *CHEK2* PV.

Germline LR variants p.I157T, p.S428F, and p.T476M are common in the general population and are frequently detected on cancer panel testing. As documented in gnomAD, a public variant database, these variants occur among 0.2% of non-Finnish European individuals, 1.1% of Ashkenazi Jewish individuals, and 0.05% of the European population.^11^ The classification of these LR variants in public repositories varies.^12,13,14,15^ Even individuals with a single *CHEK2* LR variant should be counselled on the implications of the genetic finding, as this may prompt germline testing in partners and influence family building decisions and care.

### Limitations

This study has some limitations. Despite the large scale of this study, there were still relatively few individuals with biallelic *CHEK2* variants. These data are subject to bias as these cases were ascertained through clinical cancer genetic testing. The study population is racially homogenesis (predominantly White).

Further investigation is needed to better understand how these variants act as modifiers of cancer phenotype among individuals with other genetic, familial, environmental, or lifestyle risk factors. Larger datasets are needed to determine if all 3 LR variants have the same association with breast cancer risk when combined with a *CHEK2* PV and if individuals with biallelic LR variants have no difference in their cancer risk phenotype from those with monoallelic LR variants or the general population, as our findings suggest. Commercial efforts and research groups have included p.I157T (dbSNP: rs17879961) in polygenetic risk score calculations.^16,17^ Meanwhile, p.S428F (dbSNP: rs137853011) and p.T476M (dbSNP: rs142763740) were not included in these polygenetic risk score assessments.^16,17^ Polygenetic risk scores, which included the p.I157T single-nucleotide variation, have been used to estimate breast cancer risk among those with *CHEK2* PVs^18^ and have been combined with the Tyrer-Cuzick model.^19,20^

## Conclusions

In this cohort study, biallelic *CHEK2* LR variants did not appear to have a higher cancer penetrance than was found among individuals with a single LR variant. *CHEK2* LR variants (p.I157T, p.S428F, and p.T476M) appear to be more penetrant for any cancer, multiple primary cancers, breast cancer, and bilateral breast cancer when combined with a *CHEK2* PV. Individuals with a *CHEK2* PV and an LR variant had a similar cancer phenotype to individuals with 2 PVs. These data inform the reporting of LR variants by testing laboratories and influence genetic counseling and family planning of individuals with LR variants.

## Notes

Supplement 1. **eTable 1.** Cohort Characteristics Stratified by Wild Type, *CHEK2* Variant and *CHEK2* Variant Combination **eTable 2.** Breast Cancer Subtype Stratified by Wild Type, *CHEK2* Variant and *CHEK2* Variant Combination **eTable 3.** Cancer Diagnoses Observed for Individuals With Biallelic *CHEK2* Variants

Supplement 2.
Data Sharing Statement
