# Supplementary material for: Double CHEK2 Pathogenic and Low-Risk Variants and Associated Cancer Phenotypes
Source: JAMA Netw Open. 2025 Jan 2;8(1):e2451361. doi: 10.1001/jamanetworkopen.2024.51361 (PMC11696452; doi:10.1001/jamanetworkopen.2024.51361)
Supplement: Supplement 1. — eTable 1. Cohort Characteristics Stratified by Wild Type, CHEK2 Variant and CHEK2 Variant Combination eTable 2. Breast Cancer Subtype Stratified by Wild Type, CHEK2 Variant and CHEK2 Variant Combination eTable 3. Cancer Diagnoses Observed for Individuals With Biallelic CHEK2 Variants [file jamanetwopen-e2451361-s001.pdf]

Supplementary Online Content

Bychkovsky BL, Agaoglu NB, Horton C, et al. Double *CHEK2* pathogenic and low-risk variants and associated cancer phenotypes. *JAMA Netw Open.* 2024;8(1):e2451361. doi:10.1001/jamanetworkopen.2024.51361

eTable 1. Cohort Characteristics Stratified by Wild Type, *CHEK2* Variant and *CHEK2* Variant Combination
eTable 2. Breast Cancer Subtype Stratified by Wild Type, *CHEK2* Variant and *CHEK2* Variant Combination
eTable 3. Cancer Diagnoses Observed for Individuals With Biallelic *CHEK2* Variants

This supplementary material has been provided by the authors to give readers additional information about their work.

© 2025 Bychkovsky BL et al. *JAMA Network Open.*

eTable 1. Cohort Characteristics Stratified by Wild Type, *CHEK2* Variant and *CHEK2* Variant Combination

Demographics	*CHEK2* variant category					
	**WT**<br>**33034 Pts identified**	**Single LR**<br>**1566 Pts identified**	**Single PV**<br>**2167 Pts identified**	**2 LR**<br>**13 Pts identified**	**PV+ LR**<br>**20 Pts identified**	**2 PV**<br>**21 Pts identified**
**Age at Testing, Median (IQR), y**	53 (44-63)	55 (46-64)	53 (43-62)	53 (38-66)	59 (46-65)	50 (40-58)
**Ethnicity**	**Patients, No. (%)**	**Patients, No. (%)**	**Patients, No. (%)**	**Patients, No. (%)**	**Patients, No. (%)**	**Patients, No. (%)**
African American/ Black	2247(6.8)	282 (18.0)	92 (4.2)	2 (15.4)	2 (10.0)	1(4.8)
Ashkenazi Jewish	1815 (5.5)	8 (0.5)	37 (1.7)	0 (0.0)	0 (0.0)	0 (0.0)
Asian	1582 (4.8)	17 (1.1)	66 (3.0)	0 (0.0)	0 (0.0)	0 (0.0)
Hispanic	1071 (3.2)	2 (0.1)	20 (0.9)	0 (0.0)	0 (0.0)	0 (0.0)
Other	4409 (13.4)	172 (11.0)	246 (11.4)	3 (23.1)	3 (15.0)	0 (0.0)
White	21907 (66.3)	1085 (69.3)	1706 (78.7)	8 (61.5)	15 (75.0)	20 (95.2)
**Gender**						
Female	30429 (92.1)	1434 (91.6)	1995 (92.0)	10 (76.9)	15 (75.0)	20 (95.2)
Male	2605 (7.9)	132 (8.4)	172 (7.9)	3 (23.1)	5 (25.0)	1(4.8)

Abbreviations: WT, Wildtype; Pts, Patients; LR, low-risk; PV, pathogenic or likely pathogenic variant; IQR, interquartile range

© 2025 Bychkovsky BL et al. *JAMA Network Open.*

eTable 2. Breast Cancer Subtype Stratified by Wild Type, *CHEK2* Variant and *CHEK2* Variant Combination

Characteristic	*CHEK2* variant category					
	WT*<br>30429<br>Pts identified,<br>No. (%)	Single LR<br>1434<br>Pts identified,<br>No. (%)	Single PV<br>1995<br>Pts identified,<br>No. (%)	2 LR<br>10 Pts identified,<br>No. (%)	PV+ LR<br>15<br>Pts identified,<br>No. (%)	2 PV<br>20<br>Pts identified,<br>No. (%)
**Breast cancer**	16029 (52.7)	824 (57.5)	1339 (67.1)	6 (60.0)	13 (86.7)	20 (100.0)
**ER status available**		557 (67.6)	897 (67.0)	5 (83.3)	9 (69.2)	14 (70.0)
**PR status available**		502 (60.9)	815 (60.9)	5 (83.3)	9 (69.2)	11 (55.0)
**HER2 status available**	7854 (49.0)	392 (47.6)	650 (48.5)	4 (66.7)	8 (61.5)	7 (35.0)
**ER+ (% of ER status available)**		492 (88.3)	823 (91.8)	5 (100.0)	8 (88.9)	14 (100.0)
**PR+ (% of PR status available)**		406 (80.9)	681 (83.6)	3 (60.0)	8 (88.9)	10 (90.9)
**HER2+ (% of HER2 status available)**	1492 (19.0)	73 (18.6)	165 (25.4)	3 (75.0)	2 (25.0)	2 (28.6)

Abbreviations: WT, Wildtype; Pts, Patients; LR, low-risk; PV, pathogenic or likely pathogenic variant; ER, estrogen receptor; PR, progesterone receptor; HER2, human epidermal growth factor receptor 2
*For wildtype, 78.0% (8355/10,717) of breast cancer cases were ER+ and/or PR+.

© 2025 Bychkovsky BL et al. *JAMA Network Open.*

eTable 3. Cancer Diagnoses Observed for Individuals With Biallelic *CHEK2* Variants

Genetic variant category	*CHEK2* variant 1	*CHEK2* variant 2	Gender	Personal history of cancer?	Number of cancer primaries*	Breast cancer	Bilateral or multiple breast primaries	Other cancer diagnoses	Other cancer types
2LR	c.1283C>T	c.470T>C	Female	Yes	2	Yes	Yes	No	
2LR	c.1283C>T	c.1283C>T	Female	No	0	No	No	No	
2LR	c.1283C>T	c.1283C>T	Female	Yes	1	No	No	Yes	Melanoma
2LR	c.1427C>T	c.1427C>T	Female	Yes	2	Yes	No	Yes	Thyroid
2LR	c.1427C>T	c.1427C>T	Female	No	0	No	No	No	
2LR	c.1427C>T	c.1427C>T	Female	No	0	No	No	No	
2LR	c.470T>C	c.470T>C	Female	Yes	1	Yes	No	No	
2LR	c.470T>C	c.470T>C	Female	Yes	1	Yes	No	No	
2LR	c.470T>C	c.470T>C	Female	Yes	2	No	No	Yes	Colorectal and Thyroid
2LR	c.470T>C	c.470T>C	Female	Yes	1	Yes	No	No	
2LR	c.470T>C	c.470T>C	Male	Yes	1	Yes	No	No	
2LR	c.470T>C	c.470T>C	Male	Yes	3	No	No	Yes	Kidney, Leukemia (Hairy cell) and Thyroid
2LR	c.470T>C	c.470T>C	Male	Yes	1	No	No	Yes	Kidney
PV+LR	c.1100DELC	c.470T>C	Female	Yes	1	Yes	No	No	
PV+LR	c.1100DELC	c.1283C>T	Female	Yes	2	Yes	Yes	No	
PV+LR	c.1100DELC	c.470T>C	Female	Yes	1	Yes	No	No	
PV+LR	c.1100DELC	c.1427C>T	Female	Yes	1	Yes	No	No	
PV+LR	c.1100DELC	c.470T>C	Female	Yes	1	Yes	No	No	
PV+LR	c.1100DELC	c.470T>C	Female	Yes	1	Yes	No	No	
PV+LR	c.1100DELC	c.470T>C	Female	Yes	2	Yes	Yes	No	
PV+LR	c.1100DELC	c.1283C>T	Female	Yes	2	Yes	No	Yes	Melanoma
PV+LR	c.1263DELT	c.1427C>T	Female	Yes	2	Yes	Yes	No	
PV+LR	c.444+1G>A	c.470T>C	Female	Yes	1	Yes	No	No	
PV+LR	c.444+1G>A	c.470T>C	Female	Yes	3	No	No	Yes	Lymphoma, Ovarian and Thyroid
PV+LR	c.444+1G>A	c.470T>C	Female	Yes	1	Yes	No	No	
PV+LR	c.444+1G>A	c.470T>C	Female	Yes	1	Yes	No	No	
PV+LR	c.444+1G>A	c.470T>C	Female	No	0	No	No	No	
PV+LR	EX8_9del	c.470T>C	Female	Yes	0	Yes	No	No	
PV+LR	c.1100DELC	c.1283C>T	Male	Yes	1	No	No	Yes	Hepatocellular
PV+LR	c.1100DELC	c.470T>C	Male	Yes	1	Yes	No	No	
PV+LR	c.276DELC	c.470T>C	Male	Yes	1	Yes	No	No	
PV+LR	c.444+1G>A	c.470T>C	Male	Yes	2	No	No	Yes	Colorectal and Brain cancer (Astrocytoma)
PV+LR	c.846+4_846+7DELAGTA	c.470T>C	Male	Yes	1	Yes	No	No	

Abbreviations: LR, low-risk; PV, pathogenic or likely pathogenic variant; *excluding non-melanoma skin cancers

© 2025 Bychkovsky BL et al. *JAMA Network Open.*
