# Supplementary material for: Double CHEK2 Pathogenic and Low-Risk Variants and Associated Cancer Phenotypes
Source: JAMA Netw Open. 2025 Jan 2;8(1):e2451361. doi: 10.1001/jamanetworkopen.2024.51361 (PMC11696452; doi:10.1001/jamanetworkopen.2024.51361)
Supplement: Supplement 2. — Data Sharing Statement [file jamanetwopen-e2451361-s002.pdf]

Data Sharing Statement

Bychkovsky. Double CHEK2 Pathogenic and Low-Risk Variants and Associated Cancer Phenotypes. *JAMA Netw Open*. Published January 02, 2025.
doi:10.1001/jamanetworkopen.2024.51361

Data
Data available: No

Additional Information
Explanation for why data not available: All data generated or analyzed during this study are included in this published article. We will not be sharing individual patient data as the rare genetic combinations and clinical information may make this information identifiable.
